# The Physiology of Versification—Harmonies of Organic and Animal Life

**Published:** 1875-02-01

**Authors:** Oliver Wendell Holmes


### THE PHYSIOLOGY OF VERSIFICATION—HARMONIES OF ORGANIC AND ANIMAL LIFE.

By Oliver Wendell Holmes, M. D.

*From The Boston Medical and Surgical Journal.*

WE are governed in our apparently voluntary actions by impulses derived from many obscure sources which act upon us almost without our cognizance. The digestive system legislates largely for our habits, bodily and mental, and its condition has no insignificant effect upon our intellectual and spiritual states. We are commanded to a considerable extent by our idiosyncrasies and infirmities. The secret of our diversities as social beings lies far more in our peptic capacities, in our indifference to exposure or liability to suffer from it, in our sensibility to cold and heat or to the air of ill-ventilated rooms, in the varying amount of sleep we require, in the degree of ability to bear strong light, in the quickness or dullness of our hearing, in the greater or less degree of fatigue induced by the standing posture, and in the demands of internal organs which have a will if not a voice of their own, than our friends who call us good companions or otherwise are always ready to believe.

There are two great vital movements pre-eminently distinguished by their rhythmical character,—the respiration and the pulse. These are the true time-keepers of the body; having a constant relation in health, the proportion being, as Mr. Hutchinson has shown, one inspiration to every four beats of the heart. It is very easy to prove that the first of these rhythmical actions has an intimate relation with the structure of metrical compositions. That the form of verse is conditioned by economy of those muscular movements which ensure the oxygenation of the blood is a fact which many have acted on the strength of without knowing why they did so.

Let us look first at the natural rate of respiration. Of 1817 individuals who were the subject of Mr. Hutchinson's observations, "the great majority (1731) breathed from sixteen to twenty-four times per minute. Nearly a third breathed twenty times per minute, a number which may be taken as the average." *

The "fatal facility" of the octosyllabic measure has often been spoken of, without any reference to its real cause. The reason why eight syllable verse is so singularly easy to read aloud is that it follows more exactly than any other measure the natural rhythm of respiration. In reading aloud in the ordinary way from The Lay of the Last Minstrel, from In Memoriam, or from Hiawatha, all written in this measure, the first two in iambics, or short-longs, the last in trochaics or long-shorts, it will be found that not less than sixteen nor more than twenty-four lines will be spoken in a minute, probably about twenty. It is plain, therefore, that if one reads twenty lines in a minute, and naturally breathes the same number of times during that minute, he will pronounce one line to each expiration, taking advantage of the pause

* Flint's Physiology, i, 391.

at its close for inspiration. The only effort required is that of vocalizing and articulating; the breathing takes care of itself, not even demanding a thought except where the sense may require a pause in the middle of a line. The very fault found with these octosyllabic lines is that they slip away too fluently, and run easily into a monotonous sing-song.

In speaking the ten syllable or heroic line, that of Pope's Homer, it will be found that about fourteen lines will be pronounced in the minute. If a breath is allowed to each line the respiration will be longer and slower than natural, and a sense of effort and fatigue will soon be the consequence. It will be remembered, however, that the *cæsura*, or pause in the course of the line, comes in at irregular intervals as a "breathing-place," which term is its definition when applied to music. This gives a degree of relief, but its management requires care in reading, and it entirely breaks up the natural rhythm of breathing.

The fourteen syllable verse, that of Chapman's Homer, the common metre of our hymn-books, is broken in reading into alternate lines of eight and six syllables. This also is exceedingly easy reading, allowing a line to each expiration, and giving time for a little longer rest than usual at the close of the six syllable line.

The twelve syllable line, that of Drayton's Polyolbion, is almost intolerable, from its essentially unphysiological construction. One can read the *ten* syllable line in a single expiration without any considerable effort. One instinctively divides the *fourteen* syllable line so as to accommodate it to the respiratory rhythm. But the *twelve* syllable line is too much for one expiration and not enough for two. For this reason, doubtless, it has been instinctively avoided by almost all writers in every period of our literature.

The long measure of Tennyson's Maud has lines of a length varying from fourteen to seventeen syllables, which are irregularly divided in reading for the respiratory pause. Where the sense does not require a break at some point of the line we divide it by accents, three in each half, no matter what the number of syllables; but the breaks which the sense requires so interfere with the regularity of the breathing as to make these parts of Maud among the most difficult verses to read aloud, almost as difficult as the Polyolbion.

It may be said that the law of relation here pointed out does not apply to the *writing* of verse, however it may be with regard to reading or declaiming it. But the early poems of a people are recited or sung before they are committed to writing, and even if a versifier does not read aloud as he writes, he mentally articulates every line, and takes cognizance instinctively of its physiological adjustment to respiration as he does of its smoothness or roughness, which he hears only in imagination.

The critical test of poetry by the stop-watch, and its classification according to its harmonizing more or less exactly with a great vital function, does not go very far, but it is quantitative and exactly scientific so far as it does go. The average reader will find on trial that the results given above are correct enough to justify the statements made. But here, as in astronomical observation, we must not forget the personal equation. An individual of ample chest and quiet temperament may breathe habitually only fourteen times in a minute, and find the heroic or iambic pentameter,—the verse of Pope's Homer and Gray's Elegy,—to correspond with his respiratory rhythm, and thus to be easier than any other for him to read. A person of narrower frame and more nervous habit may breathe oftener than twenty times in a minute, and find the seven syllable verse of Dyer's Grongar Hill fits his respiration better than the octosyllables of Scott or Tennyson or Longfellow. A quick-breathing little child will learn to recite verses of two and four syllables, like the story of the couple whose predilections in favor of azotized and non-azotized diet are recorded in our nursery classic, and do it easily, when it would have to catch its breath in the middle of lines of six or seven syllables.

Nothing in poetry or in vocal music is widely popular that is not calculated with strict reference to the respiratory function. All the early ballad poetry shows how instinctively the reciters accommodated their rhythm to their breathing. Chevy Chace or The Babes in the Wood may be taken as an example for verse. God save the King, which has a compass of some half a dozen notes and takes one expiration, economically used, to each line, may be referred to as the musical illustration.

The unconscious adaptation of voluntary life to the organic rhythm is perhaps a more pervading fact than we have been in the habit of considering it. One can hardly doubt that Spenser breathed habitually more slowly than Prior, and that Anacreon had a quicker respiration than Homer. And this difference, which we conjecture from their rhythmical instincts, if our conjecture is true, probably, almost certainly, characterized all their vital movements.

It seems not unlikely that other organic rhythms may be found more or less obscurely hinted at in the voluntary or animal functions. How far is *accent* suggested by or connected with the movement of the pulse, every stroke of which, if it does not lift the brain, as Bichat taught, sends a shock through its whole substance, and compresses it in its unyielding case? It is worth nothing that twenty acts of respiration mean eighty arterial pulsations, and that twenty octosyllabic lines corresponding to these eighty pulsations have exactly eighty accents. Again, there is a singular coincidence between the average pulsations of the arteries and the number of steps taken in a minute; and as we hurry our steps, the heart hurries to keep up with them. They sometimes correspond so nearly that one is reminded of the relation between the steam-chest, with its two alternately opening valves, and the piston with its corresponding movements, as we see it in the steam-engine. The doctrine of Bichat referred to above has been combated on the ground that the closely imprisoned brain could not be lifted; but the forcible impact of the four columns of arterial blood is none the less real in the normal condition than when the brain is seen to be raised through an accidental opening in the skull. So, also, notwithstanding the gradual equalization of the cardiac impulse, this impulse must be felt very extensively throughout the body. We see that it can lift a limb through a considerable space when we happen to sit with one leg crossed over the other. It is by no means impossible that the regular contractions of the heart may have obscure relations with other rhythmical movements more or less exactly synchronous with their own; that our accents and our gestures get their first impulse from the cardiac stroke which they repeat in visible or audible form. In these funeral marches which our hearts are beating, we may often keep step to the cardiac systole more nearly than our poet suspected. But these are only suggestions to be considered and tested; the relations of verse to the respiratory rhythm will be easily verified and extended by any who may care to take the trouble.

---

A Warning to Doctors is issued by the *San Francisco News Letter*, which announces its intention in future of publishing after each death-notice the name of the attending physician. — *American Medical Weekly.*

Small-Pox Epidemics. — From advices in the public prints it appears that small-pox prevails to a considerable extent in New York City. — *Med and Surg. Rep.*

